# Parents’Attitudes, Their Acceptance of the COVID-19 Vaccines for Children and the Contributing Factors in Najran, Saudi Arabia: A Cross-Sectional Survey

**DOI:** 10.3390/vaccines10081264

**Published:** 2022-08-06

**Authors:** Abdullah Ibrahim Aedh

**Affiliations:** Department of Internal Medicine, College of Medicine, Najran University, Najran 1988, Saudi Arabia; aayahya@nu.edu.sa; Tel.: +966-50-575-6913

**Keywords:** COVID-19, children, vaccine acceptance, vaccination, Saudi Arabia

## Abstract

Background: The COVID-19 pandemic is still ongoing, so it is critical to immunize the majority of people, including children, to achieve herd immunity against the pandemic. As parents are the ones who ultimately decide whether or not to vaccinate their children, this study was conducted to determine parental acceptance and hesitancy toward vaccinating their children against COVID-19, as well as their knowledge of and concerns regarding vaccination against COVID-19, as well as factors that might influence their willingness to vaccinate in Najran city, Saudi Arabia. Methods: In February 2022, a cross-sectional, questionnaire-based study using a convenientand snowball sampling technique was carried out. Parents of children between the ages of 5 and 11 were given access to an online self-administered survey. The poll was, however, open to parents of children under the age of 5. Using the Raosoft sample size calculator, the minimum necessary sample size was determined to be 384 under the assumptions of a 5% margin of error and a 95% confidence level. A *p* value of less than 0.05 was deemed significant for the statistical analysis, which was carried out using SPSS version 27. To examine the relationship between demographic factors and how drivers affect parents’ willingness to vaccinate their children against COVID-19, a chi-square test was performed. Through multivariate regression analysis, the predictors of vaccine hesitancy were identified. Results: A total of 464 responses were collected and subjected to data analysis. More than half of the parents were male (56.9%) and between the ages of 26 and 40 (56.7%). Most parents have children aged between 5–11 years (73.5%). Of parents, 72.2% showed vaccine hesitancy and were 9.5 times less likely to immunize their children against COVID-19. About 27.8% of the parents were ready to vaccinate their children against COVID-19 as soon as possible, compared to 15.51% of parents who were not at all interested in vaccinating their children. Parents under 25 (34.48%) and over 41 (37.79%), non-Saudi (40.59%), holding postgraduate and higher degrees (39.5%), earning more than 10,000 SAR per month (34.96%), working as healthcare professionals (40.36%) and in government sectors (33.93%), self-employed (33.33%), with three to five children (35.26%) and male (31.33%) demonstrated significantly high willingness to vaccinate their children against the COVID-19 vaccine compared to their counterparts. Parents who concur that COVID-19 vaccination may have serious adverse effects in children, who believe that COVID-19 is an uncommon disease and does not require vaccination, have had a family member infected withCOVID-19 with severe symptoms, who were unvaccinated and had severe to moderate symptoms after vaccination, showed significantly higher unwillingness to vaccinate their children against COVID-19. Parents who take safety precautions and do not believe that new vaccines provide an increased risk had higher vaccination intentions for their children. A positive impact of mandatory childhood vaccination was noted on the COVID-19 vaccination. Parents with children suffering from any chronic disease exhibited a 9.9 times higher hesitancy to vaccinate their children against COVID-19. A total of 47.8% of parents had come across or heard about anti-COVID-19 vaccination campaigns. A lack of adequate safety data, potential future consequences, and vaccine efficacy were the main concerns with COVID-19 vaccines. The primary information source for COVID-19-related information was the Saudi Ministry of Health (MOH). Conclusions: Parents’ hesitation to get the COVID-19 vaccine at a significant rate may compromise the success of the ongoing vaccination campaign. The development and implementation of multi-component interventions are required. Hospital- and community-based programs must be used to get in touch with parents.

## 1. Introduction

Since the initial identification of coronavirus disease-2019 (COVID-19) caused by severe acute respiratory syndrome coronavirus (SARS-CoV-2) in December 2019 in Wuhan, People’s Republic of China, the disease has spread rapidly across the world. On 11 March 2020, COVID-19 was declared a pandemic by the World Health Organization (WHO) [1]. As of 1 March 2022, over 435 million confirmed cases and over 5.9 million deaths have been reported globally and more than 745,000 confirmed cases and more than 9000 deaths have been reported from Saudi Arabia. [2]. The COVID-19 pandemic has led to an unprecedented public health crisis, stressing healthcare systems, disrupted global supply chain, transportation and affected economies [3].

Children of all ages can get COVID-19; however, children are less affected by severe disease, hospitalization and death in comparison to adults, and the majority have asymptomatic infection or mild disease [4,5,6,7,8]. The incidence of COVID-19 in children is similar to that in adults [9]. At the same time, children infected with COVID-19 are also capable of transmitting SARS-CoV-2 to others [10,11]. SARS-CoV-2 is primarily spread by way of respiratory droplets, including aerosols, and there is evidence of entry via both the lungs and the oral cavity [12]. In this context, it is imperative that even children receive effective treatment and are immunized against COVID-19 to prevent susceptibility to the disease and its further transmission.

With the advent of COVID-19, different countries across the world, including Saudi Arabia, implemented strict rules and restrictions together with social distancing, wearing of masks and travel to contain the further spread of the disease [13]. With these stricter restrictions in place, the spread of COVID-19 could be controlled to a certain extent but could not be stopped completely. In the beginning, due to the absence of effective therapies, the existing drugs were repurposed against SARS-CoV-2 and approved for the treatment of COVID-19 [14]. However, the most promising move toward curbing the spread of the COVID-19 pandemic is the inoculation of masses against SARS-CoV-2 [15,16]. Academic and research organizations and pharmaceutical industries mostly in collaboration worked hard and developed vaccines for COVID-19 and still today many vaccine candidates are in clinical trials. The development of a new vaccine is a tedious and lengthy process. However, vaccines for COVID-19 were developed at the pandemic’s speed with the help of the knowledge and expertise gained from the process of developing existing vaccines. Even in this shorter development period, all the regulatory processes were strictly followed and the safety and efficacy of vaccines was given utmost priority. The responsible health authorities and regulatory agencies keep a vigil and monitor the ongoing safety of vaccines in the real world [17,18].

After the approval of COVID-19 vaccines, immunization was rolled out in phases and prioritized for high-risk individuals, including patients with chronic and autoimmune diseases, the elderly, and healthcare workers. The COVID-19 vaccine was introduced for children aged 12 to 18 in Saudi Arabia on 25 June 2021, following the start of the vaccination campaign for the elderly and other high-risk individuals [19]. More recently, on 21 December 2021, a COVID-19 vaccination was started for children in the age group of 5 to 11 years [20]. However, the success of any vaccination program depends on the acceptance of vaccines by the target population and is important for achieving herd immunity to halt the pandemic.

The WHO defines vaccine hesitancy as a delay in the acceptance or refusal of vaccination despite the availability of vaccination services. Vaccine hesitancy is complex and context specific and varies across time, place and vaccine, and factors such as complacency, convenience and confidence influence it. The WHO has designated vaccine hesitancy as one of the most critical challenges to public health at all times [21,22]. Vaccine hesitant individuals may accept some of the vaccines while rejecting others, delay vaccinations, or accept vaccinations but have concerns [23]. The causes of vaccine hesitancy vary across different countries and across the same country. This signifies the complex and context-specific nature of vaccine hesitancy and the factors influencing it and highlights the importance of locally identifying the pertinent contributory factors of vaccine hesitancy and designing tailored interventions to tackle these [24]. The study is being conducted at a time when health authorities are implementing vaccinations for children aged 5 to 11 years. The findings would be helpful in designing and implementing newer health interventions aimed at increasing parental acceptance of the COVID-19 vaccine, as well as filling in any existing information gaps. Earlier studies conducted on a national and international scale revealed that a number of variables, such as sociodemographic characteristics, awareness of and attitudes toward COVID-19 disease and vaccines, recommended measures and vaccination, can affect acceptance of vaccination for both an individual and their children [25,26,27,28,29,30,31,32,33]. A vaccine hesitant parent may refuse or at least delay the child’s vaccination. As the decision of vaccinating children lies with their parents or guardians, it is important to study their acceptance of and attitudes toward the COVID-19 vaccine and the factors that determine vaccine acceptance and hesitancy.

To the best of my knowledge, no study conducted solely in the city or province of Najran has examined parental vaccine hesitancy to COVID-19 vaccines. Therefore, this cross-sectional study was carried out to evaluate parental behavior and attitudes toward vaccinating their children against COVID-19, adherence to safety measures, parental knowledge of and concerns regarding vaccination against COVID-19, and factors that might influence parental willingness to vaccinate their children against COVID-19.

## 2. Materials and Methods

### 2.1. Methods

This study consisted of a cross-sectional online survey and the target group was parents of children aged 5–11 years who live in the Najran city of Saudi Arabia. However, the survey was also open to parents of children under the age of 5. The study was approved by the ethical committee of Najran University with the number 43/1/22/NU/DS and was carried out in February 2022. The study was conducted according to the guidelines of the Declaration of Helsinki.

### 2.2. Setting and Participants

We distributed an online questionnaire to the parents of Najran city, Saudi Arabia. We used a convenient and snowball sampling technique to recruit the study participants. The questionnaire barcode was displayed at the entrance of shopping malls and kids’ indoor play areas and shared on social media (Facebook, Twitter, Instagram, WhatsApp status). The total population of Najran city is 344,379 [34]. Considering this, the minimum required sample size was calculated using the Raosoft sample size calculator by assuming a 5% margin of error and 95% confidence level and found to be 384. However, to represent better, we collected a larger sample of 464.

### 2.3. Development of Study Tool and Validation

The study tool was initially drafted following an extensive review of the relevant literature [25,26,27,28,29,30,31,32,33,35] to gather the most appropriate questions addressing our area of interest. Following this, the questionnaire was subjected to internal validation by experts in the fields of epidemiology, microbiology pediatrics and community health. A pilot study was conducted among 25 parents from the region to check face validity. Necessary changes were made to the questionnaire based on feedback and comments from the pilot participants. Additionally, the Cronbach’s alpha factor was calculated to ensure the reliability of the questionnaire and was found to be satisfactory (0.72).

### 2.4. Contents of theStudy Tool

#### 2.4.1. Demographic Details

Our study assessed sociodemographic characteristics, including parent’s age, gender, working area, monthly household income, nationality, marital status, religion, education and place of residence. Additionally, the following child-related characteristics, including number of children, child gender and presence of any chronic disease, were collected.

#### 2.4.2. Child Immunization History and Parents’ Source of Information Regarding COVID-19

This section assessed mandatory childhood vaccinations. Parents were asked to answer, “Have you vaccinated your child with mandatory childhood vaccines?” and were there any adverse reactions after vaccination? Parents were asked to provide the source they referred to obtain the knowledge and information about COVID-19 vaccination in children, including Ministry of Health, WHO, television (TV), media articles, social media, healthcare professionals, scientific reports, family and friends and any other. Multiple answers were made possible for this question. Similarly, parent’s concerns related to COVID-19 vaccination for their children were evaluated using statements addressing complications, lack of safety, adverse reactions, future complications of COVID-19 vaccine, etc.

#### 2.4.3. Parents’ Vaccination, Infection Status and Precautionary Measures toward COVID-19

This section assessed the parents’ COVID-19 vaccination status, whether they had taken the vaccine so far or not, how severe the symptoms were post vaccination, and whether anyone in their direct family member was infected with COVID-19 infection. Moreover, we explored the parent’s commitment in following precautionary measures related to COVID-19. These factors could directly or indirectly affect vaccine hesitancy among parents.

#### 2.4.4. Parents’ Willingness to Vaccinate Children with COVID-19 Vaccine

The willingness of parents to vaccinate their child/children with the COVID-19 vaccine was determined by asking, “Are you willing/intending to give your child/children (age group 5 to 11 years) the COVID-19 vaccine? The parents were given responses in a range of “as soon as possible, later in a few months, later in a year or more, not sure, no, but I might consider it in the future and No, never”. We considered parents who responded to give vaccination to their child “as soon as possible’ as willing to vaccinate their child, whereas all other responses were classified as unwillingness to vaccinate their child.

#### 2.4.5. Parental Attitude toward COVID-19 Vaccination in Children

Parental vaccine hesitancy was assessed using the Vaccine Hesitancy Scale (VHS), a tool created by the Strategic Advisory Group of Experts (SAGE)on Immunization of the World Health Organization (WHO) [35]. In line with Temsah et al. [25], we used a modified version of the SAGE on Immunization VHS from the WHO to measure parental vaccine hesitancy.

According to Temsah et al. [25], the statement “All childhood vaccines offered by the government program in my community are beneficial” was removed from the questionnaire to increase its relevance and better suit the study group. The nine phrases, of which six evaluated positive attitudes and three addressed parental concerns about vaccines and vaccinations, were chosen to effectively convey the need for vaccination in children. In line with Kempe et al. [36], we evaluated parental vaccine hesitancy using a 4-point Likert scale. Because recent research shows that a 4-point scale without a neutral option lowers the chance of social conformity, the option “neutral” was eliminated.

The mean parental attitude (VHS) score toward vaccines was calculated according to Temsah et al. [25] by averaging the nine items on the scale after reversing the statements that were negatively worded, with a higher score indicating a better attitude toward vaccines. Attitude VHS scores were then classified into low vs. high hesitance toward vaccines based on a cut-off value (3 points), so participants with a mean attitude score below 3 VHS attitude points were therefore considered to have high hesitance and participants with a score of ≥3 VHS points were considered to have low hesitance.

### 2.5. Data Analysis

All completed responses were subjected to completeness and consistency. SPSS version 27 was used to perform the statistical analysis, and a *p* value of less than 0.05 was considered significant. The association between demographic variables and parent’s willingness to vaccinate children against COVID-19 was analyzed using chi-square and likelihood ration tests. The impact of drivers on parent’s willingness to vaccinate children against COVID-19 was determined using a chi-square test. Multivariate regression analysis (binary logistic regression) was performed to determine the predictors of vaccine hesitancy.

## 3. Results

A total of 464 responses were collected and subjected to data analysis.

### 3.1. Demographic Details

Details regarding the sociodemographic details of the study participants are depicted in Table 1. More than half of the responding parents were male (56.9%), aged between 26–40 years (56.7%), Saudi nationals (78.2%), married (89.7%), Muslims (99.4%), residing in urban areas (90.3%), and non-healthcare (HCP) professionals (64.2%). The majority of parents has children aged between 5–11 years (73.5%) and do not suffer from any chronic disease (93.5%).

### 3.2. Parents’Willingness to Vaccinate Their Children against COVID-19

Table 2 describes the association between demographic variables and parent’s intention to vaccinate their children with COVID-19. It is apparent from the observations that parents’ age, nationality, education, monthly income, working area and working as HCP, number and gender of children and children with chronic or long-term disease have shown a significant correlation with parents’ willingness to vaccinate their children. Parents aged below 25 (34.48%) and above 41 years (37.79%) have shown significantly (*p* < 0.001) high willingness to vaccinate their children compared to parents of the 26–40 years age group. Likewise, non-Saudi parents (40.59%) showed significantly (*p* = 0.016) high intention to vaccinate their children with COVID-19. Similarly, parents holding postgraduate and higher degrees of education (39.5%), earning more than 10,000 SAR monthly income (34.96%), working as healthcare professional (40.36%), self-employed (33.33%) and working in government sectors (33.93%), having three to five (35.26%), male (31.33%) and children not suffering from any chronic disease (29.26%) demonstrated significantly high willingness to vaccinate their children against COVID-19 vaccine compared to their counterparts.

### 3.3. Vaccine Hesitancy Scale (VHS)—Attitude Regarding the Importance of Vaccinating Children against COVID-19

**Figure 1:**
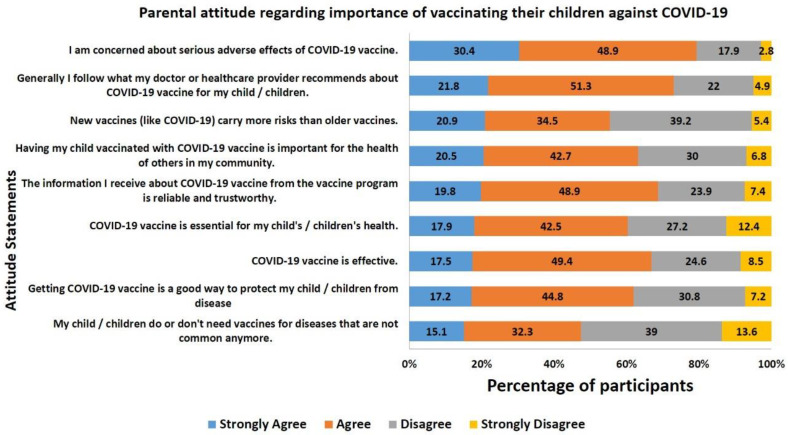
Parental attitude regarding the importance of vaccinating their children against the COVID-19.

Nine statements were incorporated to measure the adequacy of attitudes regarding the COVID-19 vaccine among parents. Parental COVID-19 vaccine hesitancy was measured using the VHS. We noticed that about one-third of parents (72.2%) had a VHS score of <3 (highly hesitant) and 27.8% had a score of >3, indicating no hesitancy toward COVID-19 vaccination for their children. The details of the responses to each of 9 attitude statements are represented in Figure 1.

More than 60% of parents have shown agreement with all six statements evaluating positive attitude, indicating that parents are aware of the COVID-19 vaccine’s importance and benefits in children. More than 70% of parents stated that they followed the child’s doctor’s recommendations regarding vaccinating their children. Nearly 70% of parents agreed that they trust and rely on information regarding COVID-19 vaccination in children. A similar percentage of parents agreed that the COVID-19 vaccine was effective. Conversely, about 40% of parents have displayed their disagreement on statements indicating “COVID-19 is essential for my child/children’s health” and “Getting the COVID-19 vaccine is a good way to protect my child/children from disease.”On the other hand, more than 40% of parents rightly disagreed with the statements “My child/children do or do not need vaccines for diseases that are not common anymore” and “New vaccines (like COVID-19) carry more risks than older vaccines.”Around 80% of the parents agreed with the statement “I am concerned about serious adverse effects of COVID-19 vaccine.”

### 3.4. Parental Behavior toward COVID-19 Vaccination, Precautionary Measures, and Childhood Vaccination

A total of 47.8% of parents have heard/seen about campaigns against COVID-19 vaccination. About half of the parents declared their commitment to following precautionary measures against COVID-19. Overall, 61.2% of parents stated having their direct family member infected with COVID-19 with mild (9.9%) to moderate symptoms (34.3%). Sadly, 0.9% of parents declared the death of family members due to COVID-19, whereas 9.3% mentioned having severe symptoms of COVID-19 infection. Almost all parents (95.9%) were vaccinated against COVID-19, with no (34.1%) or mild (31.3%) symptoms post vaccination. Altogether, 80.6% of parents declared vaccinating their children with mandatory childhood vaccination, with just no (36.6%) or mild (34.1%) symptoms related to vaccination (Table 3).

### 3.5. Parents’Concerns and Misbelieves Regarding Vaccinating Their Children with COVID-19

**Figure 2:**
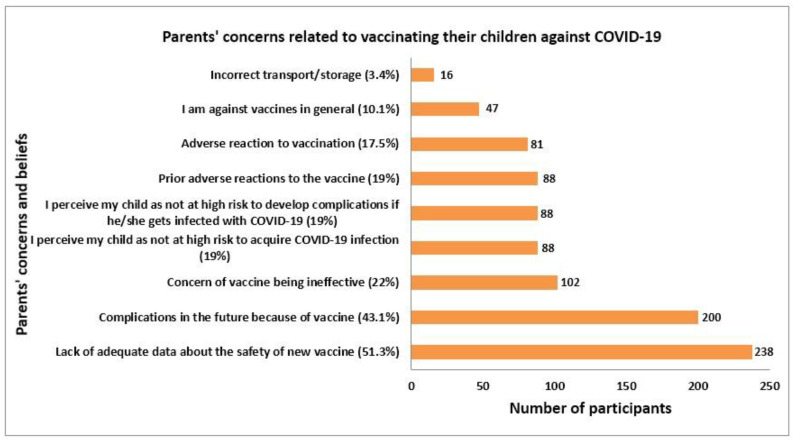
Parent’s concerns related to vaccinating their children against COVID-19.

The study participants mentioned their concerns and beliefs regarding theCOVID-19 vaccine in children, which could be a discouraging factor for them to vaccinate their children with COVID-19. More than half (51.3%) of parents stated that they had inadequate information about the safety of new vaccines in children. Likewise, 43.1% mentioned that they were worried about future complications of COVID-19 in their children. Additionally, 22% doubt the effectiveness of this vaccine in children, whereas 19% believe that their children are not at a high risk of COVID-19 infection. A negligible (3.4%) proportion of parents perceived the incorrect transportation and storage of these vaccines (Figure 2).

### 3.6. Source of COVID-19 Information

**Figure 3:**
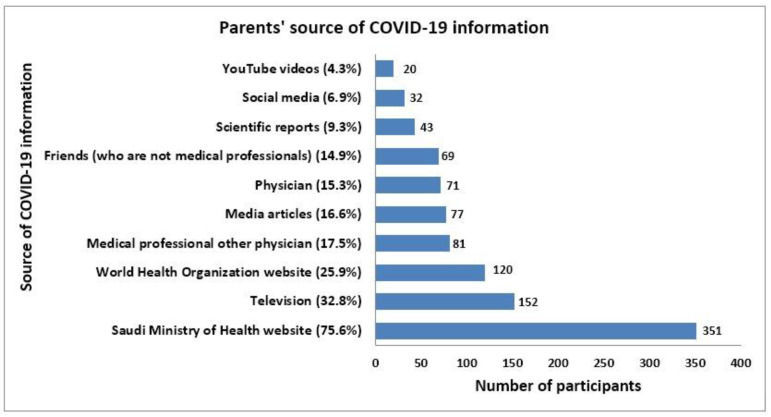
Parent’s sources of COVID-19 information.

Interestingly, 75.6% of parents used the Saudi Ministry of Health website to acquire information about COVID-19 and its vaccines, followed by TV (32.8%), the WHO website (25.9%), non-physician medical professionals (17.5%), media articles (16.6%) and physicians (15.3%). Fortunately, parents referring YouTube videos (4.3%) and social media platforms (6.9%) to obtain COVID-19-related information were negligible. About 14.9% of parents mentioned contacting friends to obtain COVID-19-related information (Figure 3).

### 3.7. Determining the Drivers of Parental Willingness to Vaccinate Their Children against COVID-19

Table 4 displays the specific drivers influencing the parent’s willingness to delay, reject or accept the COVID-19 vaccine for their children. Analysis indicated that the significant contributors to vaccine hesitancy were concern about the serious adverse effects of vaccination, rareness of the disease, and more risk of new vaccines compared to old vaccines. Furthermore, commitment toward the precautionary measures, COVID-19 infection within direct family members and severity of symptoms, parental vaccination for COVID-19 and post vaccination symptoms were the significant drivers affecting parent’s decisions regarding vaccinating their children. We noticed significantly (*p* < 0.001) higher unwillingness among parents who agree that COVID-19 vaccination may lead to serious adverse effects in their children. Similarly, parents who agreed that COVID-19 is an uncommon disease and does not need vaccination were significantly (*p* < 0.001) unwilling to vaccinate their children. Similarly, parents who disagree that their children do or do not need vaccines for diseases that are new have shown significantly (*p* < 0.001) higher intention to vaccinate their children with COVID-19. Likewise, parents who disagree that new vaccines carry more risk (*p* < 0.001) and follow precautionary measures have demonstrated high intention to vaccinate their children. Having a family member infected with COVID-19 with severe symptoms or death was significantly related to high unwillingness among parents. Unvaccinated parents and parents with severe to moderate symptoms post vaccination have shown significantly higher unwillingness toward COVID-19 vaccination for their children.

### 3.8. Multivariate Analysis to Identify the Factors Associated with Parental COVID-19 Vaccine Hesitancy for Their Children

All the variables demonstrating a significant impact in the chi-square test (age, nationality, education, family income, job sector, working as HCP, number and gender of children, presence of chronic disease, COVID-19 precautionary measures, COVID-19 infected person in direct family and earlier immunization history of child) on parents’ intention to vaccinate their children against COVID-19 were subjected to multivariate regression logistic analysis to determine the individual predictors of vaccine hesitancy among parents. The predictor selected for analysis was vaccine hesitancy. The variables including parents who are working as HCP (B: −0.993, *p*:0.007), self-employed (B: −3.633, *p*:0.040) and housewives (B: −2.423, *p*:0.011) have shown significantly negative beta coefficients, indicating that parents who are working as HCP have 0.3 times significantly higher intentions to vaccinate their children against COVID-19 compared to parents working as non-HCP. Likewise, parents who were self-employed and housewife were significantly less hesitant about COVID-19 vaccination among children. Similarly, parents of children who had received all mandatory childhood vaccinations were significantly (*p*:0.011) negatively (B:−0.899) associated with vaccine hesitancy, indicating that childhood vaccination has a positive impact on COVID-19 vaccination. Conversely, parents of the 26 to 40 year old age group demonstrated 2.16 times significantly (*p*:0.014) higher hesitancy to vaccinate their children against COVID-19 compared to parents who are 25 years and below and above 41 years. The parents with children suffering from any chronic disease demonstrated significantly (*p*:0.007) 9.9 times higher hesitancy to vaccinate children against COVID-19. Similarly, parents who had any direct family member infected with COVID-19 infection were significantly (*p*:0.048) 1.7 times hesitant to vaccinate their children. Surprisingly, parents who followed better precautionary measures against COVID-19 in somewhat (B:1.295, *p* < 0.001) and much (B:0.970, *p*:0.008) committed ways were significantly 3.6 and 2.6 times more hesitant toward vaccinating their children against COVID-19, respectively, compared to parents who followed little or no precautionary measures. Expectedly, parents with a VHS score of less than 3 points were positively (2.254) and significantly (*p* < 0.001) associated with higher vaccine hesitancy toward COVID-19 vaccines compared to parents scoring more than 3 on VHS. It was evident from the results that parents with a VHS of less than 3 were 9.5 times more hesitant toward the COVID-19 vaccination of their children (Table 5). The other variables, such as parent’s gender, nationality, education, monthly income, area of residence, and number and gender of children, have not demonstrated a significant impact on vaccine hesitancy.

## 4. Discussion

One of the most effective treatments ever, vaccinations have prevented and controlled infectious disease epidemics, helped people live longer, better lives, and saved millions of lives worldwide [37]. Herd immunity is crucial and can be attained by vaccination. Depending on biological, environmental and sociobehavioral factors, the population threshold for generating COVID-19 herd immunity ranges between 55 and 82 percent, according to a recent study [38]. However, this cannot be accomplished without immunizing large populations, including children. Additionally, the acceptance of vaccines by people for both themselves and their children is essential to the success of any immunization effort.

The findings of this study provide a wealth of information about the landscape of parental vaccine hesitancy in Najran city. According to the study, there is a relationship between parents’ sociodemographic characteristics and their willingness to vaccinate their children against COVID-19. It also discusses parents’ attitudes toward how important it is to vaccinate children against COVID-19, how they behave toward COVID-19 vaccination, precautionary measures, and mandatory childhood vaccinations, as well as their concerns and misconceptions about the importance of protecting children against COVID-19. These findings contribute important and beneficial information from this area of the region to the body of literature on parental vaccine hesitancy and will aid in the development of interventions meant to alleviate parental vaccine hesitancy.

In the present study, 72.2% of parents demonstrated vaccine hesitation with a VHS score of less than 3 and were 9.5 times less likely to immunize their children with the COVID-19. Only 27.8% of the participating parents in the current study were willing to immunize their children against COVID-19 as soon as possible, compared to 15.51% of parents who did not want to vaccinate their children at all. In Saudi Arabia, very few studies have examined parental vaccine hesitation in general or COVID-19 vaccine hesitancy in specific. In a national study, 61.9% of parents said they were hesitant to vaccinate their 5- to 11-year-old children against COVID-19, as measured by their intention to not vaccinate their children [39]. In another national survey, 45% of the participating parents were found to be hesitant toward the COVID-19 vaccine, of which only 22.3% were willing to vaccinate their children [25]. According to a study conducted at King Khalid University Hospital in Riyadh, 20% of parents are reluctant to have their children receive vaccinations, and vaccine safety is one of their top concerns [40]. A study conducted at King Abdullah University Hospital in Riyadh showed that 24.31% of the participating Saudi mothers were reluctant to vaccinate their children against COVID-19, and a similar percentage of women (24 %) planned to vaccinate their children against COVID-19 in six months [41]. In another cross-sectional survey in Saudi Arabia, 56% of parents were discovered to be unwilling to vaccinate their child against COVID-19. The acceptance of vaccination was higher among parents who intended to vaccinate themselves and who trusted the healthcare system [29].

In a Polish survey, a higher percentage of parents (44.1%) were willing to vaccinate their children as soon as possible, while 25.8% were completely against it [26]. In a study conducted in England, a significant majority of respondents (48.2%) indicated that they would unquestionably accept a COVID-19 vaccine for their children or that they were doubtful but inclined (40.9%) to do so [27]. In contrast to the results of the current study, more than 85% of mothers and mothers-to-be from India, Mexico, Colombia and Brazil said they would be willing to have their children vaccinated against COVID-19 in a survey conducted in 16 countries [28]. From three national surveys conducted in Saudi Arabia, little less than half of the parents (47.6%, 44% and 38.1%) indicated that they would be willing to vaccinate their children against COVID-19 [25,29,39]. Another multi-regional study conducted in Saudi Arabia found that 51.67% of parents were favorable about vaccinating their children [30].

Parents’ hesitation to vaccinate their children is known to be influenced by a variety of factors, which can change over time, across countries, and even within a single region, including sociodemographic traits, cultural and religious beliefs, political beliefs, and economic beliefs [42,43].

Similar to the results of a national survey in Saudi Arabia, which found that parents in older age groups were more likely to accept COVID-19 vaccination of their children [25], parents in the current study were more willing to vaccinate their children if they were 25 years of age or younger and 41 years of age or older. Similar to the results of this study, a different study indicated that parents between the ages of 31 and 40 were somewhat less likely to vaccinate their children against COVID-19 [39]. Similar to the findings of Temsah et al. [25], the current study did not find any evidence of a significant association between parents’ gender and their intention to vaccinate their children with the COVID-19 vaccine; however, in a study from Poland [26], mothers demonstrated favorable attitudes and were willing to vaccinate their children as soon as possible with the COVID-19 vaccine. In contrast, females were more hesitant than males to vaccinate their children, according to a Saudi national study of parents of children aged 5 to 11 years [39].

In the current study, non-Saudi parents showed a considerably high intention to vaccinate their children with the COVID-19 vaccine, in contrast to the results of two national surveys conducted in Saudi Arabia [25,29]. In a national survey by Almalki et al. [39], married participants were found to be more hesitant to vaccinate their children than single participants, and in a survey by Ennaceur and Al-Mohaithef [29], married parents were found to be more willing to accept their children receiving the COVID-19 vaccine than parents who were living separately, divorced or widowed; however, in the current study, marital status had no significant impact on parents’ willingness to vaccinate the children.

Multiple studies have assessed the impact of parents’ educational standing on their level of hesitation and willingness to consent to vaccinations for their children. In the present study, parents with post-graduate and higher degrees were more inclined to vaccinate their children, in contrast to the findings of Temsah et al. [25] and Almalki et al. [39]. The results are in line with those of Ennaceur and Al-Mohaithef [29], who found that parents with graduate and post-graduate education were more likely than parents with only a high school diploma or less to approve their children receiving the COVID-19 vaccine. Additionally, research has also shown that parents with higher education levels are around four times more likely than parents with lower education levels to have concerns about the safety of vaccines [44].

Similar to the findings of earlier studies, parents in my study who earned more money each month (more than 10,000 SAR) had a greater intention to vaccinate their children with the COVID-19 vaccine. As per a 2018 survey, the average monthly household income in Najran province was 8697 SAR (one SAR equals 0.27 U.S. dollars) [45]. According to a study done in Illinois, USA, parents with yearly household incomes over USD 150,000 had lower COVID-19 VH scores than parents with annual household incomes under USD 40,000 [31]. In England, participants with lower household incomes were 1.8 times more likely than parents with medium family incomes to oppose giving their children the COVID-19 vaccine [27]. Additionally, similar to the findings of Temsah et al. [25], HCPs had higher intentions of vaccinating their children against COVID-19. This could be explained by the fact that HCPs are aware of the value of vaccinations, as well as the characteristics and severity of COVID-19.

It is recognized that a person’s willingness to take precautions can affect their decision to vaccinate themselves and their children. Similar to the findings from prior studies, in the current investigation, individuals with families who had a high level of dedication to precautionary measures against COVID-19 had higher intentions of vaccinating their children against COVID-19 than those with little to no commitment. COVID-19 preventive measures were discovered to be a significant predictor of parents’ acceptance of the COVID-19 vaccine in a study by Skjefte et al. [28]. In a different study, parents who reported less compliance with social distancing practices also reported less acceptance of the COVID-19 vaccination and vice versa [46].

In accordance with the findings of the study by Almalki et al. [39], parents in the current study who had a family member infected with COVID-19 demonstrated a high unwillingness to give their children the COVID-19 vaccination. An adult household member’s history of COVID-19 infection was revealed to be a predictor of parental VH in a study by Almalki et al. [39]. The conclusions of Saudi Arabia’s other two national surveys, however, were quite different. In a study by Temsah et al. [25], parents who had at least one immediate family member with a history of COVID-19 infection were more likely to vaccinate their children against COVID-19.In a study by Ennaceur and Al-Mohaithef [29], parents who were worried about getting COVID-19 infection for themselves or a member of their family were more likely to vaccinate their children. In the current study, the severity of COVID-19 in afflicted family members was another predictor of parents’ willingness to vaccinate their children with the COVID-19 vaccine; however, a study by Temsah et al. [25] found no evidence of such an impact. Contrary to the Polish study [26], the severity of COVID-19 in children was related to their likelihood of receiving vaccination.

Similar to Saudi Arabia’s large-scale national studies, parents who had already received the COVID-19 vaccination themselves were less hesitant and more willing to vaccinate their children. A child’s vaccination history is also related to the likelihood that the child will receive the COVID-19 vaccine. Similar to the findings of Almalki et al. [39], the current study found that routine childhood vaccinations had a favorable effect on parents’ intentions to vaccinate their children with COVID-19 vaccine and significantly reduced vaccine hesitancy. Additionally, a previous study discovered that parents whose children had received all of their recommended vaccines were more receptive to an expedited COVID-19 shot [47].

Parents’ sources of information about COVID-19 vaccination should also be investigated and examined since these can have a big impact on parents’ attitudes toward the vaccine and their decision to vaccinate their children. Numerous studies have emphasized the significance of the sources used. The findings of Temsah et al. [25], who found that 85.9% had referred to the Saudi Ministry of Health (MOH) for information and were found to be significantly more intending to vaccinate their children against COVID-19, are almost identical to the findings of the majority of the parents (75.6%), who used the Saudi MOH as their source for information regarding COVID-19 vaccination. The WHO website was used as a reliable source of information by 25.9% of parents, less when compared with findings obtained in the study by Temsah et al. [25], wherein 43.4% had referred to it but were found to be significantly less predicted to vaccinate their children against COVID-19. With 52%of respondents considering these as a source of information and 48% expressing confidence in the same, respondents in the US also had the highest level of confidence in government entities [31]. Parents who had high levels of trust in the healthcare system (69.6%) were more likely to vaccinate their children against COVID-19 than those who had low levels of trust (51.1%), according to a poll by Ennaceur and Al-Mohaithef [29].

Social media (6.9%) and YouTube videos (4.3%) were used as sources of information on COVID-19 by very few parents in the current study, suggesting that they were aware of the abundance of misleading information available on these platforms. This is in contrast to the findings of Temsah et al. [25], which showed that 35.4% of parents used social media platforms and 14.4%of parents watched YouTube videos. In the United States (US) [31] and Poland [26], 67% and 78.6% of parents, respectively, used the internet as a resource for information on COVID-19 vaccines. Only 26% of parents who reported utilizing the internet as a source of information expressed faith in it in the US, where about 40% of participants used social media as a reference [31]. The internet, YouTube and social networking platforms should all be avoided, and even when utilized, only the accounts of organizations trusted and acknowledged by the healthcare industry or of the industry itself should be used.

Interestingly, 32.8% and 16.6% of parents in my study reported obtaining knowledge about COVID-19 from television and media articles, respectively. The media may be quite important in obtaining the necessary and accurate information. Healthcare organizations and independent researchers should use the national media as a platform to run campaigns, tell the targeted population about COVID-19 and promote COVID-19 vaccination. The acceptance of vaccines among parents can be considerably increased with the aid of the national media.

Parents’ decisions regarding vaccination can also be significantly influenced by HCPs and the high-quality information and content published in media reports and scientific studies. In Poland, parents who sought advice from a doctor or read scientific studies were more willing to get their children vaccinated as soon as possible [26]. In the US as well, 40% of the parents utilized their HCPs for information and 44% showed confidence in them and were significantly associated with lower odds of VH [31]. In a study conducted in 16 countries, it was discovered that pregnant women were more likely to vaccinate themselves or their unborn children than non-pregnant women were −45.9% versus 54.6% [28]. Family pediatricians, in particular, can have a significant impact on parents’ decisions regarding their children’s vaccination. An Italian study discovered that pediatricians’ recommendations to fully vaccinate children had a significant impact on parents’ decisions about vaccination. Family pediatricians and other doctors were the most consulted and trusted sources of information among both pro-vaccine and hesitant parents [48]. HCPs should employ the best communication methods when starting a conversation on vaccination. Parents who favor vaccinations and those who are apprehensive about vaccinations can both benefit from a good communication plan that allays their concerns and fears. HCPs should be able to make effective, powerful and clearer recommendations to parents who are reluctant about vaccination. HCPs working in vaccination programs can enhance both parents’ and children’s overall immunization experiences. Since public sector primary health care centers are where most childhood vaccination services are provided, the HCPs employed by these facilities can have a significant impact on raising vaccine acceptance and favorably influencing parents’ choices.

Parents’ hesitation and acceptance of COVID-19 vaccines for children may be influenced by a range of concerns about vaccinating their children, particularly when it comes to the COVID-19 vaccines. The major concern cited by parents in the present study was the lack of adequate data about the safety of the new vaccine (51.3%), corresponding to the findings of Temsah et al. [25], wherein about 69% of parents cited it as a concern. In England, 68% of participants reported insufficient evidence due to a rushed vaccination [27]. A national survey in Saudi Arabia of parents of children aged 5 to 11 found that 48.3% of those who initially refused to vaccinate their children would do so if they were given adequate information about the COVID-19 vaccine [39]. In Poland, 56% felt that the preparations had not been adequately tested [26]. In the current study, 43.1% reported potential vaccine-related complications as a significant concern, while 22% listed vaccine effectiveness as a concern. Additionally, 17.5% expressed concern about vaccination’s side effects. Other investigations have come to similar conclusions. Parents who expressed reservations about the safety or effectiveness of the COVID-19 vaccines were more likely to be hesitant to vaccinate their children, according to a national survey conducted in Saudi Arabia among parents of children aged 5 to 11 [39]. One of the main predictors of parents’ reluctance to COVID-19 immunization in Poland was their concerns about potential future problems, which were voiced by 51.3% of parents [26]. Another factor that was discovered to be a predictor of parental concern was the effectiveness of the preparation, and more than 50% of the parents who had doubts about effectiveness stated that they would never get their children immunized [26]. In studies by Temsah et al. [25] and Ennaceur and Al-Mohaithef [29], 60.6% and 22.9% of parents, respectively, listed vaccine side effects as one of their major concerns, while 22.9% in the latter study [29] also mentioned vaccine effectiveness as a concern. Concerns regarding safety were cited by 62.1%of participants in England as one of the main barriers to taking the vaccine for children [27]. In a poll carried out in 16 countries, 32.7% of mothers stated that they wanted to see more information on the safety and efficacy of COVID-19 immunizations among children, and 28.4% believed that the vaccine was unsafe and might have negative side effects [28]. In a multi-regional survey conducted in Saudi Arabia, 31.43% cited side-effect concerns and 65.44% cited vaccine effectiveness as the main justifications for declining to vaccinate their children against COVID-19 [30]. In the current study, fewer parents (19%) believed their child was not at a high risk of contracting COVID-19 infection, and an equal number believed their child would not experience complications if they contracted COVID-19, findings that are quite similar to those of Temsah et al. [25]. In a national study conducted in Saudi Arabia, it was discovered that parents of children aged 5 to 11 who thought their children’s general health was very poor were the ones who were most hesitant to vaccinate them against COVID-19 [39]. In England, one of the participants’ (19%) believed that children hardly ever contract COVID-19 was cited as a cause for not accepting the vaccine for children [27]. In a multi-regional study conducted in Saudi Arabia, 34.07%of parents thought acquiring immunity after contracting COVID-19 was preferable to receiving vaccination [30].

Another factor contributing to vaccine hesitancy among parents and posing a severe threat to children’s health is anti-vaccination campaigns that disseminate inaccurate, twisted, false and misleading information about viruses and vaccinations. Anti-vaccination rhetoric thrives on social media in particular, so it is important to keep an eye on it. To immunize more people against COVID-19 and so safeguard them and the community at large, anti-vaccination groups should be closely watched and actively suppressed [49,50].

The majority of studies have shown that the same factors that affect parental vaccine hesitancy also affect VH in diverse populations as a whole. About 91.9% of undergraduate students in central and southern Italy were keen to get vaccinated against COVID-19. It was found that prior influenza vaccination and knowledge of the COVID-19 vaccine were both associated with acceptance of the vaccination against COVID-19 [51]. In a survey conducted among adults 65 years and above from southern Italy, a satisfactory level of knowledge about COVID-19 and the associated control measures was revealed in the enrolled sample [52]. In a different Italian survey of elderly people in southern Italy, 92.7% of participants had received a COVID-19 vaccination or were willing to do so; nevertheless, less than half of the sample was in favor of vaccinations and agreed with mandatory immunization. A higher educational level was found to be positively correlated with acceptance of the COVID-19 vaccination. For COVID-19, about 61.1% of older persons consulted healthcare professionals and scientists, whereas 38.6% obtained their knowledge from the media (television and magazines) [53]. In a Saudi Arabian survey of older persons aged 50 and over, 43.85 % said that they would be open to receiving the COVID-19 vaccination when it became available. Older men were more likely than older women to be open to vaccination. The willingness to receive vaccinations was highly correlated with high levels of education. The likelihood of older persons receiving the COVID-19 vaccine was lower if they had previously rejected any vaccination. People who showed high or very high levels of fear about contracting the disease were more inclined to receive the COVID-19 vaccination. The most often mentioned causes for vaccine hesitation were adverse side effects (27.01%) and safety and effectiveness concerns (22.63%) [54]. In a separate study of the general population in the southwest of Saudi Arabia, 52.79% said they would be open to receiving the COVID-19 vaccine and nearly 64 % agreed that getting the vaccine is necessary to protect oneself and that the vaccine is safe, efficient and effective. Acceptance of the COVID-19 vaccine was significantly predicted by perceived COVID-19 risk, past history of seasonal influenza vaccination, and trust in the healthcare system [55]. A systematic review and meta-analysis found that older age, trust in COVID-19 vaccines, and fear of COVID-19 were predictors of COVID-19 vaccination uptake, whereas distrust in the government and concerns about the safety and side effects of COVID-19 vaccines were causes of dilapidated vaccination in pregnant women. The overall percentage of expectant mothers who received the COVID-19 vaccine was 27.5% [56].

However, despite parental vaccine hesitancy, which has been noted regionally and globally in several studies [25,26,27,28,29,30,31,32,33,39,40,41], by September 2021, more than 90% of 12- to 18-year-old students were immunized against COVID-19 [57], and as of 19 June 2022, a total of 66,700,629 vaccine doses were given in Saudi Arabia [58]. These results offer a glimmer of hope and optimism that parents will eventually vaccinate their children against COVID-19, if not right away, then at the very least soon.

Suggestions: In order to address and combat vaccine hesitancy among parents, special techniques and multi-component interventions should be created and put into place. Children should receive the COVID-19 vaccine as a requirement. When addressing child vaccinations with parents, it is critical to get started as early as possible and take a presumptuous stance. Parents should be effectively informed of the significance and necessity of vaccinating children against COVID-19. It is important to address parents’ concerns about vaccination, particularly those related to safety, potential side effects and vaccine efficacy. Parents should be made aware of the rigorous processes used throughout the development and testing of vaccines on humans, the part that various international and regional health authorities, including the SFDA, have played in evaluating the safety and efficacy data and the ongoing post-marketing surveillance that is carried out. The public and parents should be informed promptly, correctly and effectively of any new information regarding the safety and effectiveness of COVID-19 vaccines in the pediatric population. Information should be distributed through trusted platforms and sources. HCPs who are involved in vaccination campaigns should be empathetic and share their own experiences caring for patients with their parents. These can assist in fostering a relationship of trust with the parents and persuade them to vaccinate their children against COVID-19.

In different areas of Saudi Arabia and at different times, surveys evaluating vaccine hesitancy against COVID-19 among parents should be conducted. The results of these surveys should be taken into account when designing future interventions. Interventions such as poster messages promoting COVID-19 vaccines, educational fact sheets from health organizations, vaccine information pamphlets, school immunization policies, reminder/recall systems using telephone calls, short message service (SMS), mailed letters, emails, etc. should be utilized and evaluated for their effectiveness in curbing parental vaccine hesitancy.

Training HCPs should be prioritized, with a focus on developing their counseling abilities and fostering confidence in the public healthcare system. Finally, it is important to act sternly with those who propagate inaccurate and misleading information regarding COVID-19 vaccines.

Limitations: This study has a number of limitations. The study’s cross-sectional design represents parents’ responses at a particular time. It is hard to track the final decision made by parents regarding the COVID-19 vaccination of their children. As more vaccine evidence becomes available and vaccination efforts are undertaken for children under the age of five, parents’ hesitance toward and acceptance of vaccination may change in the future. As the study was limited to the city of Najran, its conclusions cannot be generalized to include the full province of Najran, other surrounding provinces or the entire Kingdom of Saudi Arabia. The use of an online survey for the study, which limited participation to individuals with internet access, may have resulted in selection bias and mindless responses from participants who were only trying to finish the survey, which resulted in the collection of inaccurate and erroneous responses. Despite the increasing efforts made to reach a larger number of individuals, only those who were interested would have willingly taken part in the survey. The problems with internet connectivity and device-related issues may have led to a surge in errors or parents giving up on the survey altogether. It is unclear how the non-completion group and the survey-completion group varied from one another. Estimating the response rate was not possible due to the design of the study and the online questionnaire. The parents who were questioned might not be representative of Najran city, which is still another limitation.

## 5. Conclusions

Parents showed a significant rate of hesitancy to the COVID-19 vaccine, which may jeopardize the success of the ongoing vaccination drive. The key concerns were a lack of adequate safety data, potential future consequences and vaccine effectiveness. To increase parental acceptance of COVID-19 vaccines, the MOH and other pertinent institutions should effectively convey safety and efficacy data for COVID-19 vaccines generated in the future. In addition, the majority of parents obtained their knowledge of COVID-19 vaccines from the MOH website. The findings of this study provide insight into the landscape of parental vaccine hesitancy and its impact on Najran City. They will also assist healthcare professionals, regulatory agencies, and healthcare organizations in creating future interventions aimed at reducing parental vaccine hesitancy.

## Figures and Tables

**Table 1:** Demographic details of the study participants.

Demographic Information *n* = 464			
Characteristics	Category	Number (*n*)	Percentage (%)
Age	25 years and below	29	6.3
	26 to 40 years	263	56.7
	41 years and above	172	37.1
Gender	Male	264	56.9
	Female	200	43.1
Nationality	Saudi	363	78.2
	Non-Saudi	101	21.8
Marital Status	Married	416	89.7
	Divorced or separated	48	10.3
Religion	Muslim	461	99.4
	Non-Muslim	3	0.6
Place of Residence	Urban	419	90.3
	Rural	45	9.7
Parents Education	High school or less	188	40.5
	College Degree	157	33.8
	Postgraduate or higher	119	25.6
Family’s Monthly Income	Less than 5000 SR	92	19.8
	5000 to 10,000 SR	111	23.9
	more than 10,000 SR	123	26.5
	Prefer not to answer	138	29.7
Are you a Health-care professional?	Yes	166	35.8
	No	298	64.2
Job sector	Government	221	47.6
	Private	71	15.3
	Self-Employed	3	0.6
	Unemployed	16	3.4
	Retired	16	3.4
	Housewife	121	26.1
	Student	16	3.4
Number of Children	two or less	165	35.6
	3 to 5	224	48.3
	6 to 8	68	14.7
	More than 8	7	1.5
Children of 5–11 years age	Yes	341	73.5
	No	123	26.5
Gender of children	All male	150	32.3
	All female	87	18.8
	Both male and female	227	48.9
Children with chronic diseases?	Yes	30	6.5
	No	434	93.5

**Table 2:** Association between demographic variables and parents’ willingness to vaccinate their children with COVID-19 vaccines.

Variables	Sub-Group	Willingness to Vaccinate Children against COVID-19						
		Yes, as Soon as Possible (%)	Delaying for Few Month (%)	Delaying for Year and Above (%)	Undecided (%)	No, I Might Consider in Future (%)	No, Never (%)	*p* Value
**Age**	25 years and below	10 (34.48)	7 (24.14)	3 (10.34)	8 (27.59)	1 (3.45)	Zero	<0.001 *
	26 to 40 years	54 (20.53)	33 (12.55)	15 (5.70)	93 (35.36)	25 (9.51)	43 (16.35)	
	41 years and above	65 (37.79)	25 (14.53)	12 (6.98)	27 (15.70)	14 (8.13)	29 (16.86)	
**Gender**	Male	83 (31.44)	37 (14.02)	17 (6.44)	65 (24.62)	19 (7.20)	43 (16.29)	0.257
	Female	46 (23)	28 (14)	13 (6.5)	63 (31.5)	21 (10.5)	29 (14.5)	
**Nationality**	Saudi	88 (24.24)	52 (14.33)	24 (6.61)	105 (28.93)	30 (8.26)	64 (17.63)	0.016 *
	Non-Saudi	41 (40.59)	13 (12.87)	6 (5.94)	23 (22.77)	10 (9.9)	8 (7.92)	
**Marital Status**	Married	112 (26.92)	59 (14.08)	27 (6.49)	112 (26.92)	37 (8.89)	69 (16.59)	0.397
	Divorced or separated	17 (35.42)	6 (12.5)	3 (6.25)	16 (33.33)	3 (6.25)	3 (6.25)	
**Religion**	Muslim	129 (27.98)	64 (13.88)	29 (6.29)	127 (27.55)	40 (8.68)	72 (15.62)	0.328
	Non-Muslim	Zero	1 (33.33)	1 (33.33)	1 (33.33)	Zero	Zero	
**Place of Residence**	Urban	119 (28.4)	60 (14.32)	26 (6.21)	117 (27.92)	34 (8.11)	63 (15.04)	0.636
	Rural	10 (22.22)	5 (11.11)	4 (8.89)	11 (24.44)	6 (13.33)	9 (20)	
**Parents Education**	High school or less	52 (27.66)	28 (14.89)	16 (8.51)	56 (29.79)	12 (6.38)	24 (12.77)	<0.001 *
	College Degree	30 (19.11)	16 (10.19)	9 (5.73)	53 (33.76)	21 (13.38)	28 (17.83)	
	Postgraduate or higher	47 (39.5)	21 (17.65)	5 (4.2)	19 (15.97)	7 (5.88)	20 (16.81)	
**Family’s Monthly Income**	Less than 5000 SR	22 (23.91)	12 (13.04)	8 (8.7)	33 (35.87)	5 (5.43)	12 (13.04)	<0.001 *
	5000 to 10,000 SR	21 (18.92)	5 (4.5)	11 (9.91)	30 (27.03)	16 (14.41)	28 (25.23)	
	more than 10,000 SR	43 (34.96)	23 (18.7)	6 (4.88)	26 (21.14)	7 (5.69)	18 (14.63)	
	Prefer not to answer	43 (31.16)	25 (18.12)	5 (3.62)	39 (28.26)	12 (8.7)	14 (10.14)	
**Are you a Health-care professional?**	Yes	67 (40.36)	29 (17.47)	9 (5.42)	36 (21.69)	10 (6.02)	15 (9.04)	<0.001 *
	No	62 (20.81)	36 (12.08)	21 (7.05)	92 (30.87)	30 (10.07)	57 (19.13)	
**Job sector**	Government	75 (33.93)	28 (12.66)	11 (4.97)	53 (23.98)	24 (10.85)	30 (13.57)	0.001 *
	Private	14 (19.71)	10 (14.08)	5 (7.04)	20 (28.16)	9 (12.67)	13 (18.3)	
	Self-Employed	1 (33.33)	1 (33.33)	Zero	Zero	Zero	1 (33.33)	
	Unemployed	4 (25)	Zero	5 (31.25)	1 (6.25)	1 (6.25)	5 (31.25)	
	Retired	Zero	4 (25)	Zero	8 (50)	Zero	4 (25)	
	Housewife	31 (25.61)	17 (14.04)	8 (6.61)	40 (33.05)	6 (4.95)	19 (15.7)	
	Student	4 (25)	5 (31.25)	1 (6.25)	6 (37.5)	Zero	Zero	
**Number of children**	Two or less	38 (23.03)	23 (13.93)	13 (7.87)	46 (27.87)	25 (15.15)	20 (12.12)	<0.001 *
	3 to 5	79 (35.26)	24 (10.71)	13 (5.8)	64 (28.57)	11 (4.91)	33 (14.73)	
	6 to 8	11 (16.17)	14 (20.58)	4 (5.88)	17 (25)	4 (5.88)	18 (26.47)	
	More than 8	1 (14.28)	4 (57.14)	Zero	1 (14.28)	Zero	1 (14.28)	
**Children of 5–11 years age**	Yes	91 (26.68)	49 (14.36)	16 (4.69)	100 (29.32)	29 (8.5)	56 (16.42)	0.107
	No	38 (30.89)	16 (13)	14 (11.38)	28 (22.76)	11 (8.94)	16 (13)	
**Gender of children**	All male	47 (31.33)	21 (14)	12 (8)	25 (16.66)	13 (8.66)	32 (21.33)	0.003 *
	All female	16 (18.39)	11 (12.64)	7 (8.04)	37 (42.52)	4 (4.59)	12 (13.79)	
	Both male and female	66 (29.07)	33 (14.53)	11 (4.84)	66 (29.07)	23 (10.13)	28 (12.33)	
**Children with chronic diseases?**	Yes	2 (6.66)	6 (20)	1 (3.33)	13 (43.33)	1 (3.33)	7 (23.33)	0.040 *
	No	127 (29.26)	59 (13.59)	29 (6.68)	115 (26.49)	39 (8.98)	65 (14.97)	

* *p* < 0.05.

**Table 3:** Parental behavior, vaccination related to COVID-19, and immunization history of their children.

Parental Behavior, Immunization and Precautionary Measures Related to COVID-19			
	Participant’s Response	Frequency	Percentage (%)
Have you ever heard about or seen the campaign against COVID-19 vaccination (anti-vaccination movements)?	Yes	222	47.8
	No	242	52.2
Describe your family’s commitment to the precautionary measures against the COVID-19?	No commitment	10	2.2
	Little commitment	47	10.1
	Somewhat commitment	180	38.8
	Much committed	128	27.6
	Great deal of commitment	99	21.3
Did anyone within your direct family get infected with COVID-19?	Yes	284	61.2
	No	180	38.8
How severe were the symptoms of the infected person(s)?	Very mild/asymptomatic	15	3.2
	Mild	46	9.9
	Moderate	159	34.3
	Severe	43	9.3
	Very severe	17	3.7
	Death	4	0.9
	No COVID-19 infection	180	38.8
As a parent did you take the COVID-19 vaccine?	Yes	445	95.9
	No	19	4.1
Did you have any adverse reactions after vaccination?	Mild	145	31.3
	Moderate	106	22.8
	Severe	55	11.9
	No	158	34.1
Child/Children’s immunization history			
Have you vaccinated your child with mandatory childhood vaccines?	Yes	374	80.6
	No	90	19.4
Adverse reactions after vaccination in child?	Mild	158	34.1
	Moderate	95	20.5
	Severe	41	8.8
	No	170	36.6

**Table 4:** Drivers of parental willingness to vaccinate their children against COVID-19.

Driver	Responses		Intention to Vaccinate Child against COVID-19				
		Total (%)	Yes	Delay	Undecided	No	*p* Value
**I am concerned about serious adverse effects of COVID-19 vaccine.**	Strongly agree	141 (30.38)	23(16.31)	10 (7.09)	39 (27.65)	69 (48.93)	<0.001 *
	Agree	227 (48.92)	56 (24.66)	61 (26.87)	78 (34.36)	32 (14.09)	
	Disagree	83 (17.88)	41 (49.39)	23 (27.71)	11 (13.25)	8 (9.63)	
	Strongly disagree	13 (2.8)	9 (69.23)	1 (7.69)	Zero	3 (23.07)	
**My child/children do or don’t need vaccines for diseases that are not common anymore.**	Strongly agree	70 (15.08)	11 (15.71)	10 (14.28)	14 (20)	35 (50)	<0.001 *
	Agree	150 (32.32)	28 (18.66)	23 (15.33)	49 (32.66)	50 (33.33)	
	Disagree	181 (39)	57 (31.49)	48 (26.51)	57 (31.49)	19 (10.49)	
	Strongly disagree	63 (13.57)	33 (52.38)	14 (22.22)	8 (12.69)	8 (12.69)	
**New vaccines (like COVID-19) carry more risks than older vaccines.**	Strongly agree	97 (20.9)	7 (7.21)	10 (10.3)	26 (26.8)	54 (55.67)	<0.001 *
	Agree	160 (34.48)	41 (25.62)	30 (18.75)	51 (31.87)	38 (23.75)	
	Disagree	182 (39.22)	70 (38.46)	53 (29.12)	44 (24.17)	15 (8.24)	
	Strongly disagree	25 (5.38)	11 (44)	2 (8)	7 (28)	5 (20)	
**Describe your family’s commitment to the precautionary measures against the COVID-19?**	No commitment	10 (2.15)	4 (40)	2 (20)	Zero	4 (40)	<0.001 *
	little commitment	47 (10.12)	13 (27.65)	3 (6.38)	20 (42.55)	11 (23.4)	
	Somewhat commitment	180 (38.79)	36 (20)	51 (28.33)	55 (30.55)	38 (21.11)	
	Much committed	128 (27.58)	37 (28.9)	29 (22.65)	38 (29.68)	24 (18.75)	
	Great deal of commitment	99 (21.33)	39 (39.39)	10 (10.1)	15 (15.15)	35 (35.35)	
**Did anyone within your direct family get infected with COVID-19?**	Yes	284 (61.2)	63 (22.18)	61 (21.47)	81 (28.52)	79 (27.81)	0.005 *
	No	180 (38.79)	66 (36.66)	34 (18.88)	47 (26.11)	33 (18.33)	
**How severe were the symptoms of the infected person(s)?**	Very mild	15 (3.23)	3 (20)	2 (13.33)	4 (26.66)	6 (40)	0.032 *
	Mild	46 (9.91)	12 (26.08)	9 (19.56)	11 (23.91)	14 (30.43)	
	Moderate	159 (34.26)	39 (24.52)	37 (23.27)	44 (27.67)	39 (24.52)	
	Severe	43 (9.26)	8 (18.6)	8 (18.6)	12 (27.9)	15 (34.88)	
	Very severe	17 (3.66)	1 (5.88)	5 (29.41)	6 (35.29)	5 (29.41)	
	Death	4 (0.86)	Zero	Zero	4 (100)	Zero	
	No COVID-19 infection	180 (38.79)	66 (36.66)	34 (18.88)	47 (26.11)	33 (18.33)	
**As a parent did you take the COVID-19 vaccine?**	Yes	445 (95.9)	129 (28.98)	90 (20.22)	125 (28.08)	101 (22.69)	0.001 *
	No	19 (4.09)	Zero	5 (26.31)	3 (15.78)	11 (57.89)	
**Did you have any adverse reactions after vaccination?**	Mild	145 (31.25)	41 (28.27)	39 (26.89)	42 (28.96)	23 (15.86)	<0.001 *
	Moderate	106 (22.84)	31 (29.24)	21 (19.81)	29 (27.35)	25 (23.58)	
	Severe	55 (11.85)	7 (12.72)	5 (9.09)	15 (27.27)	28 (50.9)	
	No	158 (34.05)	50 (31.64)	30 (18.98)	42 (26.58)	36 (22.78)	
**Have you vaccinated your child with mandatory childhood vaccines?**	Yes	374 (80.6)	111 (29.67)	80 (21.39)	100 (26.73)	83 (22.19)	0.080
	No	90 (19.39)	18 (20)	15 (16.66)	28 (31.11)	29 (32.22)	

* *p* < 0.05.

**Table 5:** Identifying the variables associated with parental intention to vaccinate their children.

Independent Variables	Variable Coefficient(B)	*p*-Value	OR (95% CI)Adjusted *
*Parental Vaccine Hesitancy against COVID-19 (YES)*			
Age			
25 years and below	−0.782	0.337	0.457 (0.092–2.262)
26 to 40 years	0.773	0.014 *	2.165 (1.167–4.019)
41 years and above	-	-	1.00
**Parent Working as Healthcare Professional**			
Yes	−0.993	0.007 *	0.370 (0.181–0.758)
No	-	-	1.00
**Job sector**			
Government	−1.693	0.058	0.184 (0.032–1.059)
Private	−0.896	0.344	0.408 (0.064–2.606)
Self employed	−3.633	0.040 *	0.026 (0.001–0.844)
Unemployed	−1.224	0.244	0.294 (0.038–2.303)
Retired	17.565	0.998	0.354 (0.0562–2.135)
Housewife	−2.423	0.011 *	0.089 (0.014–0.578)
Student	-	-	1.00
**Children suffering from chronic disease**			
Yes	2.295	0.007 *	9.922 (1.895–51.939)
No	-	-	1.00
**Family’s commitment to follow** COVID-19 **precautionary measure**			
No commitment	−0.577	0.525	0.562 (0.095–3.326)
Little commitment	0.690	0.181	1.993 (0.726–5.470)
Somewhat commitment	1.295	<0.001 *	3.653 (1.781–7.492)
Much committed	0.970	0.008 *	2.637 (1.284–5.417)
Great deal of commitment	-	-	1.00
**COVID-19 infected person in family**			
Yes	0.531	0.048 *	1.701 (0.989–2.926)
No	-	-	1.00
**Mandatory childhood Immunization**			
Yes	−0.899	0.011 *	0.407 (0.204–0.813)
No	-	-	1.00
**Vaccine Hesitancy score (VHS)**			
Hesitant VHS score < 3 points	2.254	<0.001 *	9.529 (5.972–15.204)
Not Hesitant: VHS score ≥ 3 points	-	-	1.00

* *p* < 0.05.

## Data Availability

Data are available from the author for researchers who meet the criteria to access confidential data.
